# Prevalence of *mcr-1* in Colonized Inpatients, China, 2011–2019

**DOI:** 10.3201/eid2709.203642

**Published:** 2021-09

**Authors:** Cong Shen, Lan-Lan Zhong, Zhijuan Zhong, Yohei Doi, Jianzhong Shen, Yang Wang, Furong Ma, Mohamed Abd El-Gawad El-Sayed Ahmed, Guili Zhang, Yong Xia, Cha Chen, Guo-Bao Tian

**Affiliations:** Sun Yat-sen University Zhongshan School of Medicine, Guangzhou, China (C. Shen, L.-L. Zhong, M.A.E.-G.E.-S. Ahmed, G. Zhang, G.-B. Tian);; Sun Yat-sen University Key Laboratory of Tropical Diseases Control, Guangzhou (C. Shen, L.-L. Zhong, M.A.E.-G.E.-S. Ahmed, G. Zhang, G. Tian);; Guangzhou University of Chinese Medicine The Second Clinic Medical College, Guangzhou (C. Shen, C. Chen);; The Second Affiliated Hospital of Guangzhou University of Chinese Medicine, Guangdong Provincial Hospital of Traditional Chinese Medicine, Guangzhou (C. Shen, C. Chen);; Sun Yat-Sen University The Fifth Affiliated Hospital, Zhuhai, China (Z. Zhong);; University of Pittsburgh School of Medicine, Pittsburgh, Pennsylvania, USA (Y. Doi);; Fujita Health University School of Medicine, Aichi, Japan (Y. Doi);; China Agricultural University, College of Veterinary Medicine, Beijing, China (J. Shen, Y. Wang);; China Agricultural University, College of Animal Science and Technology, Beijing (J. Shen, Y. Wang);; Third Affiliated Hospital of Guangzhou Medical University, Guangzhou (F. Ma, Y. Xia);; Misr University for Science and Technology, Cairo, Egypt (M.A.E.-G.E.-S. Ahmed);; Xizang Minzu University School of Medicine, Xianyang, China (G. Tian)

**Keywords:** *mcr-1*, colistin, prevalence, microbial, drug resistance, antimicrobial resistance, China

## Abstract

In response to the spread of colistin resistance gene *mcr-1*, China banned the use of colistin in livestock fodders. We used a time-series analysis of inpatient colonization data from 2011–2019 to accurately reveal the associated fluctuations of *mcr-1* that occurred in inpatients in response to the ban.

Heavy use of antimicrobials in agricultural, human, and veterinary applications correlates directly with emergence and spread of antimicrobial resistance, thereby threatening the effective management of clinical infections (*1*,*2*). An example of this association is the global dissemination of the antimicrobial resistance gene (ARG) *mcr-1*, conferring resistance to the last-line antimicrobial drug colistin. The *mcr-1* gene has been prevalent in ecosystems that use colistin as a growth promoter in food-producing animals, as seen in China before 2017 (*2*–*5*).

To counteract the high prevalence of *mcr-1* and align with One Health principles, the government in China formally banned colistin as an animal feed additive on April 30, 2017 (*6*). Previous research demonstrated that colistin resistance rates and *mcr-1* prevalence in *Escherichia coli* from human and animal samples declined substantially in China, according to a regional study conducted in Guangzhou during 2015–2019 (p<0.0001). These data suggest the effectiveness of colistin stewardship in reducing colistin resistance in both livestock and humans (*4*,*5*). However, the sampling strategy of these studies was limited to evaluating only several cross-sectional timepoints from before and after the ban, resulting in uncertainty about the exact timing of the effect.

To characterize the complete prevalence dynamics of human *mcr-1* colonization, including the periban period, we constructed a 9-year monthly time series for April 2011–December 2019, over which time 13,630 fecal samples from colonized inpatients were previously taken, by further evaluating *mcr-1* prevalence of 3,823 stored fecal samples collected during April–September 2016, January–September 2017–2018, and January–December 2019. We combined these data with those from our previous studies (*3*,*5*) (Appendix Table 1). We used a 3-month moving average approach to remove noise and substituted missing data for 7 months of the time series by using the mean values of the 2 months flanking any month with missing data (Appendix). Through changepoint analysis (Appendix) (*7*), we identified 5 changepoints, dividing the time series into 6 periods (Figure).

**Figure:**
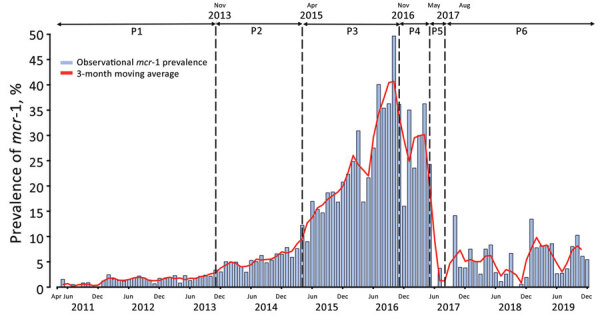
Time series of monthly *mcr-1* prevalence in colonized inpatients, China, April 2011–December 2019. The *mcr-1* prevalence was recorded each month in observed data (blue histogram) and 3-month moving average data (solid red line). Vertical dashed lines indicate significant changepoints identified in the changepoint analysis: November 2013, May 2015, November 2016, May 2017, and August 2018. The government of China formally banned colistin as an animal feed additive on April 30, 2017. P, time period.

We observed that *mcr-1* prevalence in human fecal samples was low (<3%) in the early period, before October 2013, demonstrating that the *mcr-1* gene was circulating to a limited extent in human populations before late 2013 in period 1 (P1). We observed a significant increase in *mcr-1* colonization prevalence after November 2013 in period 2 (P2) that lagged behind increases of *mcr-1* prevalence observed in livestock from 2011 (*2*) and was consistent with dissemination from this reservoir. The third period (P3) showed a sharp increase in *mcr-1* human colonization prevalence, followed by a peak in October 2016, suggesting that *mcr-1* was rapidly spreading in human settings, potentially attributable to an extremely high *mcr-1* prevalence (>60%) in livestock around the time (*4*,*5*,*8*). Beginning in November 2016, in period 4 (P4), pilot decreases in colistin use as an animal feed additive were already being implemented (*4*) before the complete ban in 2017. We observed declines in human *mcr-1* colonization prevalence during this period that were temporally consistent with declines in *mcr-1* prevalence observed in livestock (*8*). The fifth period (P5) showed a dramatic decline in human *mcr-1* colonization prevalence, correlating with the complete ban of colistin in animal feed (*6*). The rapid impact of this intervention is indicative of the dramatic effect that curtailing a selection pressure can have in constraining ARG prevalence and could be a template for combatting other ARGs. In the last period evaluated, period 6 (P6), *mcr-1* prevalence fluctuated at a low level (monthly average 5.3%), in accordance with the *mcr-1* prevalence observed in healthy human carriers, pigs, and chickens after the colistin ban (*5*). Alhough currently at low levels, *mcr-1* prevalence should be monitored continually to detect any signs of its resurgence, particularly given that colistin was approved for human clinical use in China in January 2017 (*9*).

In conclusion, we characterized the dynamic landscape of *mcr-1* over a 9-year period in China and found that colistin stewardship interventions in livestock were reflected in the *mcr-1* prevalence in human fecal colonization samples within a month of a large-scale, national ban on colistin usage. Partial reductions in colistin use beginning in November 2016 rapidly reduced the *mcr-1* prevalence and turned around the alarming increases observed during 2015–2016. The complete ban implemented on April 30, 2017, significantly and immediately reduced *mcr-1* prevalence to near pre-2015 levels. Of interest, however, the background *mcr-1* prevalence in 2019 was still higher than that observed during 2011–2013, perhaps associated with the approval of colistin for human clinical use in China in January 2017 (*9*). As a result of our findings, we strongly encourage interdisciplinary surveillance involving clinicians, veterinary specialists, and environmentalists to further investigate and evaluate changes in ARG prevalence across different human, animal, and environmental niches to improve holistic understanding of the impact and timeframe of different stewardship interventions.

Appendix Additional information about the prevalence of *mcr-1* in colonized inpatients, China, 2011–2019.
