# Supplementary material for: Prevalence of mcr-1 in Colonized Inpatients, China, 2011–2019
Source: Emerg Infect Dis. 2021 Sep;27(9):2502–4. doi: 10.3201/eid2709.203642 (PMC8386786; doi:10.3201/eid2709.203642)
Supplement: Appendix — Additional information about the prevalence of mcr-1 in colonized inpatients, China, 2011–2019. [file 20-3642-Techapp-s1.pdf]

Article DOI: https://doi.org/10.3201/eid2709.20-3642

Prevalence of *mcr-1* in Colonized Inpatients, China, 2011–2019

Appendix

Data collection

To investigate the longitudinal dynamics of *mcr-1* prevalence in *mcr-1*-colonized patients, we retrospectively analyzed 13,630 samples from April 2011 to December 2019 monthly in Guangzhou, China, except for the months of January 2012, February 2013, February 2014, March 2017, February 2018, and March and April in 2019, for which samples/data were not available. Prevalence data from April 2011 to March 2016 (except January 2012, February 2013, and February 2014 as above), were acquired from our previous study (*1*), in which 8,022 human fecal samples were obtained from three tertiary hospitals in Guangzhou, China. Among them, 497 samples were identified as *mcr-1*-positive.

The prevalence data from October to December in 2016–2018 were obtained from another previous study (*2*), in which we identified 364/2395 *mcr-1*-positive human fecal samples from two hospitals which were also sites in the study cited above (*1*).

mcr-1 prevalence data from April to September in 2016–2018 and from January to December in 2019 were obtained additionally for this study (except for data from February 2018, March and April in 2019, missing - as above). We collected 3823 samples from human colonization samples in two hospitals. The sampling hospitals and the inclusion criteria are consistent with above study (*2*).

Sample collection, sample culture, *mcr-1* screening for the samples in this study

Sampling procedures and *mcr-1* identification were consistent with our previous studies (*1*,*2*). Briefly, human fecal samples were collected using rectal swabs from inpatients without any diagnosed infections. All samples were cultured on non-selective nutrient broth media overnight at 37°C. Total DNA was extracted using the boiling method and screened for *mcr-1* by polymerase chain reaction (PCR) as previously described.[2] *mcr-1*-positive samples were re-cultured on selective agar (MacConkey + colistin [2 mg/L]) and incubated for 18–24 hours, 37°C). Subsequently, up to 10 Enterobacteriaceae colonies were sub-cultured to MacConkey agar with colistin (2 mg/L). The samples were identified as positive for *mcr-1* if any one colony was positive for *mcr-1* by PCR.

Time series analysis

Missing data for Jan 2012, February 2013, February 2014, March 2017 and February 2018 were substituted by the mean values of the two relevant flanking months. The missing data of March and April in 2019 were substituted by the mean values of February and May in 2019. A 3-month moving average (MA) approach was used to remove noise.

We conducted a changepoint detection analysis to identify the time points of significant changes in the monthly overall *mcr-1* prevalence from April 2011 to December 2019. For this analysis, we used the *cpt.meanvar* function from the changepoint package and explored a variety of penalty values and methods (*3*), retaining the most consistently identified changepoints with the proposed pruned exact linear time (PELT) algorithm. As a result, we identified five changepoints in the time series, which was divided into six periods [period 1 (April 2011 to November 2013), period 2 (December 2013 to April 2015), period 3 (May 2015 to November 2016), period 4 (December 2016 to May 2017), period 5 (June 2017 to August 2017), period 6 (September 2017 to December 2019)] (Figure 1).

Appendix Table. Data underpinning the combined *mcr-1* prevalence monthly time series in human fecal colonization samples from Guangzhou, China, April 2011-December 2019.

Period	Date	Sample size	*mcr-1*-positive sample	*mcr-1* prevalence % (95%CI)	Three-month moving average %	Data reference
1	Apr 2011	138	0	0	/	Zhong LL, et al. Clin Infect Dis. 2018; 66: 676–85.
	May 2011	66	1	1.52 (0–4.46)	0.51	
	Jun 2011	144	0	0	0.67	
	Jul 2011	201	1	0.5 (0–1.47)	0.17	
	Aug 2011	183	0	0	0.44	
	Sep 2011	122	1	0.82 (0–2.42)	0.56	
	Oct 2011	232	2	0.86 (0–2.05)	0.56	
	Nov 2011	168	0	0	0.29	
	Dec 2011	39	0	0	0.41	
	Jan 2012	N.D.	N.D.	1.22	1.22	
	Feb 2012	82	2	2.44 (0–5.78)	1.75	
	Mar 2012	63	1	1.59 (0–4.67)	1.77	
	Apr 2012	156	2	1.28 (0–3.05)	1.35	
	May 2012	85	1	1.18 (0–3.47)	1.31	
	Jun 2012	202	3	1.49 (0–3.15)	1.49	
	Jul 2012	166	3	1.81 (0–3.83)	1.83	
	Aug 2012	183	4	2.19 (0.07–4.3)	1.93	
	Sep 2012	102	0	1.8	1.80	
	Oct 2012	213	3	1.41 (0–2.99)	1.28	
	Nov 2012	155	1	0.65 (0–1.91)	1.25	
	Dec 2012	117	2	1.71 (0–4.06)	1.32	
	Jan 2013	62	1	1.61 (0–4.75)	1.76	
	Feb 2013	N.D.	N.D.	1.94	1.94	
	Mar 2013	88	2	2.27 (0–5.39)	1.67	
	Apr 2013	125	1	0.8 (0–2.36)	1.78	
	May 2013	88	2	2.27 (0–5.39)	1.45	
	Jun 2013	156	2	1.28 (0–3.05)	1.74	
	Jul 2013	181	3	1.66 (0–3.52)	1.69	
	Aug 2013	141	3	2.13 (0–4.51)	2.05	
	Sep 2013	127	3	2.36 (0–5)	2.20	
	Oct 2013	189	4	2.12 (0.06–4.17)	2.61	
2	Nov 2013	209	7	3.35 (0.91–5.79)	2.83	Zhong LL, et al. Clin Infect Dis. 2018; 66: 676–85.
	Dec 2013	166	5	3.01 (0.41–5.61)	3.79	

Period	Date	Sample size	*mcr-1*-positive sample	*mcr-1* prevalence % (95%CI)	Three-month moving average %	Data reference
	Jan 2014	80	4	5 (0.22–9.78)	4.33	
	Feb 2014	N.D.	N.D.	4.97	4.97	
	Mar 2014	182	9	4.95 (1.79–8.09)	4.67	
	Apr 2014	147	6	4.08 (0.88–7.28)	3.99	
	May 2014	102	3	2.94 (0–6.22)	4.10	
	Jun 2014	133	7	5.26 (1.47–9.06)	4.41	
	Jul 2014	159	8	5.03 (1.63–8.43)	5.51	
	Aug 2014	128	8	6.25 (2.06–10.4)	5.36	
	Sep 2014	167	8	4.79 (1.55–8.03)	5.45	
	Oct 2014	188	10	5.32 (2.11–8.53)	5.55	
	Nov 2014	168	11	6.55 (2.81–10.9)	6.12	
	Dec 2014	108	7	6.48 (1.84–11.12)	6.96	
	Jan 2015	102	8	7.84 (2.63–13.06)	6.74	
	Feb 2015	68	4	5.88 (0.29–11.47)	7.11	
	Mar 2015	79	6	7.59 (1.75–13.44)	8.56	
	Apr 2015	131	16	12.21 (6.61–17.82)	9.59	
3	May 2015	156	14	8.97 (4.49–13.46)	12.71	Zhong LL, et al. Clin Infect Dis. 2018; 66: 676–85.
	Jun 2015	118	20	16.95 (10.18–23.72)	13.77	
	Jul 2015	117	18	15.38 (8.85–21.92)	15.67	
	Aug 2015	177	26	14.69 (9.47–19.9)	16.24	
	Sep 2015	161	30	18.63 (12.62–24.65)	17.38	
	Oct 2015	218	41	18.81 (13.62–23.99)	18.08	
	Nov 2015	238	40	16.81 (12.06–21.56)	18.79	
	Dec 2015	183	38	20.77 (14.89–26.64)	19.97	
	Jan 2016	103	23	22.33 (14.29–30.37)	22.64	
	Feb 2016	137	34	24.82 (17.58–32.05)	26.01	
	Mar 2016	123	38	30.89 (22.73–39.06)	24.18	
	Apr 2016	297	50	16.84 (12.58–21.09)	23.11	This study
	May 2016	236	51	21.61 (16.36–26.86)	21.98	
	Jun 2016	349	96	27.51 (22.82–32.19)	29.73	
	Jul 2016	327	131	40.06 (34.75–45.37)	34.32	
	Aug 2016	407	144	35.38 (30.74–40.03)	37.23	
	Sep 2016	262	95	36.26 (30.44–42.08)	40.43	
	Oct 2016	278	138	49.64 (43.76-55.52)	40.68	Shen C, et al. The Lancet Microbe. 2020;1: e34–43.
4	Nov 2016	404	149	36.14 (31.1-41.18)	33.93	

Period	Date	Sample size	*mcr-1*-positive sample	*mcr-1* prevalence % (95%CI)	Three-month moving average %	Data reference
	Dec 2016	99	16	16.16 (8.91-23.41)	29.05	Shen C, et al. The Lancet Microbe. 2020;1: e34–43.
	Jan 2017	78	27	34.62 (24.06-45.18)	24.85	This study
	Feb 2017	68	17	25 (14.71-35.29)	29.52	
	Mar 2017	N.D.	N.D.	30.63	29.93	
	Apr 2017	80	29	36.25 (25.72-46.78)	30.18	
5	May 2017	173	42	24.28 (17.89–30.67)	20.18	This study
	Jun 2017	44	0	0	9.33	
	Jul 2017	54	2	3.7 (0–8.74)	1.23	
6	Aug 2017	57	0	0	1.23	This study
	Sep 2017	20	0	0	4.72	
	Oct 2017	106	15	14.15 (7.51-20.79)	6.02	Shen C, et al. The
	Nov 2017	277	11	3.92 (1.63-6.21)	7.29	Lancet Microbe.
	Dec 2017	246	9	3.66 (1.6–6.53)	5.07	2020;1: e34–43.
	Jan 2018	40	3	7.5 (0–15.66)	5.44	This study
	Feb 2018	N.D.	N.D.	5.03	5.03	
	Mar 2018	78	2	2.56 (0–6.07)	5.03	
	Apr 2018	40	3	7.5 (0–15.66)	6.13	
	May 2018	60	5	8.33 (1.34–15.33)	6.23	
	Jun 2018	70	2	2.86 (0–6.76)	4.10	
	Jul 2018	90	1	1.11 (0–3.28)	2.18	
	Aug 2018	39	1	2.56 (0–7.52)	3.45	
	Sep 2018	90	6	6.67 (1.51–11.82)	3.08	
	Aug 2018	62	0	0	2.41	Shen C, et al. The
	Nov 2018	175	1	0.57 (0–1.69)	0.83	Lancet Microbe.
	Dec 2018	138	3	2.17 (0–4.6)	5.31	2020;1: e34–43.
	Jan 2019	67	9	13.43 (5.27–21.6)	7.71	This study
	Feb 2019	90	7	7.78 (2.24–13.31)	9.8	
	Mar 2019	N.D.	N.D.	8.17	8.04	
	Apr 2019	N.D.	N.D.	8.17	8.31	
	May 2019	70	6	8.57 (2.01–15.13)	6.47	
	Jun 2019	113	3	2.65 (0–5.62)	4.66	
	Jul 2019	73	2	2.74 (0–6.48)	3	
	Aug 2019	83	3	3.61 (0–7.63)	4.78	
	Sep 2019	25	2	8 (0–18.63)	7.29	
	Oct 2019	39	4	10.26 (0.73–19.78)	8.12	

Period	Date	Sample size	*mcr-1*-positive sample	*mcr-1* prevalence % (95%CI)	Three-month moving average %	Data reference
	Nov 2019	82	5	6.1 (0.92–11.28)	7.25	
	Dec 2019	222	12	5.41 (2.43–8.38)	/	

References

1. Zhong LL, Phan HTT, Shen C, Vihta KD, Sheppard AE, Huang X, et al. High rates of human fecal carriage of *mcr-1*-positive multidrug-resistant Enterobacteriaceae emerge in China in association with successful plasmid families. Clin Infect Dis. 2018;66:676–85. PubMed https://doi.org/10.1093/cid/cix885

2. Shen C, Zhong LL, Yang Y, Doi Y, Paterson D, Stoesser N, et al. Dynamics of *mcr-1* prevalence and *mcr-1*-positive *Escherichia coli* after the cessation of colistin use as a feed additive for animals in China: a prospective cross-sectional and whole genome sequencing-based molecular epidemiological study. Lancet Microbe. 2020;1:e34–43. https://doi.org/10.1016/S2666-5247(20)30005-7

3. Killick R, Eckley I. Changepoint: an R package for changepoint analysis. J Stat Softw. 2014;58:1–19. https://doi.org/10.18637/jss.v058.i03
